# Fibroblast Growth Factor 21: A More Effective Biomarker Than Free Fatty Acids and Other Insulin Sensitivity Measures for Predicting Non-alcoholic Fatty Liver Disease in Saudi Arabian Type 2 Diabetes Patients

**DOI:** 10.7759/cureus.50524

**Published:** 2023-12-14

**Authors:** Suhad Bahijri, Basmah Eldakhakhny, Sumia Enani, Ghada Ajabnoor, Alaa S Al-Mowallad, Lubna Alsheikh, Amani Alhozali, Aliaa A Alamoudi, Anwar Borai, Jaakko Tuomilehto

**Affiliations:** 1 Department of Clinical Biochemistry, King Abdulaziz University Faculty of Medicine, Jeddah, SAU; 2 Saudi Diabetes Research Group, Deanship of Scientific Research, King Abdulaziz University, Jeddah, SAU; 3 Food, Nutrition and Lifestyle Unit, King Fahd Medical Research Centre, King Abdulaziz University, Jeddah, SAU; 4 Department of Food and Nutrition, Faculty of Human Sciences and Design, King Abdulaziz University, Jeddah, SAU; 5 Department of Biochemistry, King Abdulaziz University, Jeddah, SAU; 6 Department of Internal Medicine, King Abdulaziz University Hospital, Jeddah, SAU; 7 King Abdullah International Medical Research Center (KAIMRC), King Saud Bin Abdulaziz University for Health Sciences, Jeddah, SAU; 8 Department of Public Health, University of Helsinki, Helsinki, FIN; 9 Public Health Promotion Unit, Finnish Institute for Health and Welfare, Helsinki, FIN

**Keywords:** non-alcoholic fatty liver disease (nafld), type 2 diabetes, fgf-21, quicki, fibroblast growth factor 21

## Abstract

Background

Non-alcoholic fatty liver disease (NAFLD) is more prevalent among individuals with type 2 diabetes (T2DM), elevating their risk of cardiovascular diseases (CVDs) and premature mortality. There is a need to modify treatment strategies to prevent or delay these adverse outcomes. Currently, there are no sensitive or specific biomarkers for predicting NAFLD in Saudi T2DM patients. Therefore, we aimed to explore the possibility of using fibroblast growth factor 21 (FGF-21), free fatty acids (FFAs), homeostatic model assessment for insulin resistance (HOMA-IR), and quantitative insulin sensitivity check index (QUICKI) as possible markers.

Methodology

In this study, a total of 67 T2DM patients were recruited. NAFLD was detected by ultrasonography in 28 patients. Plasma glucose, FFAs, FGF-21, and serum insulin were measured in fasting blood samples. HOMA-IR and QUICKI were calculated. The means of the two groups with and without NAFLD were statistically compared. The receiver operating characteristics (ROC) curve and the area under the curve (AUC) were used to assess the ability to identify NAFLD.

Results

The mean levels of FGF-21 and HOMA-IR were significantly higher and that of QUICKI was significantly lower in patients with NAFLD than in those without (p < 0.001, p = 0.023, and p = 0.018, respectively). FGF-21 had the highest AUC to identify NAFLD (AUC = 0.981, 95% confidence interval = 0.954-1, P < 0.001). The AUCs for HOMA-IR, QUICKI, and FFA were <0.7. The highest sensitivity, specificity, positive likelihood ratio, and the lowest negative likelihood ratio were found when FGF-21 was used to predict NAFLD.

Conclusions

FGF-21 may be used as a biomarker to predict NAFLD in people with T2DM due to its high sensitivity and specificity compared to the other markers.

## Introduction

Non-alcoholic fatty liver disease (NAFLD) is defined as ≥5% hepatic fat content in the absence of excessive consumption of alcohol or use of certain pharmacotherapy that could induce steatosis, viral infection, or other chronic liver diseases [1,2]. NAFLD includes a range of pathological conditions, ranging from simple steatosis to non-alcoholic steatohepatitis (NASH), cirrhosis, and hepatocellular carcinoma [3].

A meta-analysis of studies published in 2016 found that NAFLD was the most common cause of chronic liver disease, with a global prevalence of about 25% [4]. However, its prevalence has increased to 30%, as reported in a more recent systemic review; hence, it is considered the leading cause of liver-related morbidity and mortality [5]. In Saudi Arabia, the total NAFLD prevalence was estimated at 8,451,000 cases (25.7%) in 2017, with cases with steatosis only representing 83.6% of all NAFLD cases, and a projected increase of the total to 31.7% by 2030 [6].

NAFLD is considered the hepatic manifestation of metabolic syndrome (MetS), with obesity, insulin resistance (IR), and some other components of the syndrome among its known risk factors [7].

NAFLD is more common in people with type 2 diabetes mellitus (T2DM) [3,8], increasing their risk of developing cardiovascular disease [9,10], as well as the risk of premature mortality [11], and requiring modification to management strategy to avoid or delay these complications. In Saudi Arabia, NAFLD is suspected in patients, especially those with T2DM, presenting with no specific symptoms, accompanied by abnormal liver tests or hepatomegaly, and is usually detected by ultrasound [12]. However, such advanced equipment is not available in public healthcare centers, and those suspected to have NAFLD are usually referred to larger hospitals, which could be in other areas, thus, delaying the diagnosis and management of the disease.

From the above, it is evident that early diagnosis of NAFLD is needed, especially in people with T2DM. Indeed, there is an urgent need for sensitive biomarkers that can be measured in smaller health centers lacking the more advanced equipment needed for diagnosis.

In our recent research, we investigated the potential of using specific biological and biochemical markers, along with readily available indices, to identify significant associations with NAFLD in individuals with T2DM to pinpoint those who would benefit from advanced and costly diagnostic tests for confirmation. We employed body mass index (BMI), waist circumference (WC), serum fasting insulin levels, triglycerides, and liver enzymes in various equations to determine the Fatty Liver Index, Hepatic Steatosis Index, NAFLD Liver Fat Score, and the Triglycerides and Glucose Index [13]. However, all investigated indices showed low specificity for predicting NAFLD. Therefore, there is still a need to identify novel markers to improve sensitivity and specificity for NAFLD in people with T2DM in primary care.

Fibroblast growth factor 21 (FGF-21) appears to be a likely candidate for investigation as it has been reported to be engaged in the inter-organ endocrine signaling axes, which are pertinent for the maintenance of the entire-body homeostasis as they govern the metabolism and homeostasis [14,15]. Indeed, increased serum FGF-21 levels have been associated with diabetes, obesity, and MetS [16,17]. In addition, FGF-21 is reportedly engaged in the actions of various antidiabetic agents [18,19]. Furthermore, key risk factors for NAFLD, including insulin insensitivity, obesity, and dyslipidemia, are alleviated by FGF-21, and FGF-21 has been reported to reverse liver steatosis while counteracting obesity and enhancing insulin sensitivity [20]. These findings suggest that FGF-21 may be upregulated under the NAFLD condition and may be involved in protecting from the progression of NAFLD by reversing steatosis and enhancing the metabolic energy status.

Moreover, the accumulation of triglycerides in the liver in people with NAFLD has been reported to be associated with various causes, including increased de novo lipogenesis [21,22], increased lipolysis in the adipocytes, increased delivery of free fatty acids (FFAs) to the liver [21,23], and decreased lipid clearance consequent to impaired fatty acid oxidation and lower lipid secretion [24,25]. Hence, the plasma level of FFAs is a possible marker worthy of investigation as increased lipolysis is suggested as one of the causes of hepatic triglyceride accumulation in NAFLD. This is thought to be a result of increased IR resulting in increased hormone-sensitive lipase (HSL) activity in the adipocytes. This leads to increased release of FFAs and increased FFA flux to the liver, hence, increased synthesis of triglycerides, without an increase in their export [26]. Furthermore, as T2DM is characterized by IR, which is one of the risk factors for NAFLD, investigating the relationship between measures of IR such as homeostatic model assessment for insulin resistance (HOMA-IR) and quantitative insulin sensitivity check index (QUICKI) as possible predictors of NAFLD seems logical, especially as it has not been studied before in the Saudi population.

Therefore, this study measured plasma levels of FGF-21, circulating levels of FFAs, HOMA-IR, and QUICKI in people with T2DM and without NAFLD. The aim was to determine specific and sensitive markers that may be used to predict this condition in Saudi people with T2DM.

## Materials and methods

Participants and study design

The study design was outlined in detail earlier [13], summarized as follows: T2DM patients were recruited from the outpatient endocrine clinics at King Abdulaziz University Hospital in a cross-sectional study design from April 1, 2015, until March 31, 2016. The study was approved by the Committee on the Ethics of Human Research at the Faculty of Medicine, King Abdulaziz University, Jeddah, Saudi Arabia (approval number: No-61-15). Written consent was obtained from all participants. Diabetic patients with hemochromatosis, viral hepatitis, Wilson’s disease, primary biliary cirrhosis, autoimmune hepatitis, impaired renal function, sclerosing cholangitis, biliary obstruction, ischemic cardiac or cerebrovascular disease, alpha-1 antitrypsin deficiency, malignancies, or alcohol consumption were excluded from the study. A predesigned questionnaire, including sociodemographic information, medical history, and drugs used, was completed in a face-to-face interview. Blood pressure (BP) was measured using standardized techniques [27]. Anthropometric measurements were taken by following the standard methods and using standardized equipment, and BMI was calculated.

A sensitive abdominal ultrasound machine (ACUSON X300™ ultrasound system, premium edition (PE) by Siemens, New York, NY, USA) was used to screen for NAFLD. Based on the results, participants were categorized into cases (those with NAFLD) and controls (those without NAFLD).

Plasma fasting glucose and serum insulin were measured in the Clinical Chemistry Laboratory at the National Guard Hospital, King Abdul-Aziz Medical City in Jeddah. Plasma glucose was measured spectrophotometrically using an Abbott Architect c8000 autoanalyzer (Abbott, Illinois, USA). Serum insulin measurement was performed on an Abbott Architect i2000 autoanalyzer (Abbott, Illinois, USA) using a chemiluminescent microparticle immunoassay method.

Serum FFA levels were measured using an enzymatic colorimetric method assay for non-esterified fatty acids (Wako Diagnostics USA Corporation). Serum FGF-21 levels were determined using an enzyme-linked immunosorbent assay (FGF-21, UNQ3115/PRO10196; BioVendor LLC, Asheville, North Carolina, USA). Both FFA and FGF-21 were measured manually at the Food, Nutrition, and Lifestyle Research Unit at King Fahd Medical Research Centre.

The HOMA-IR index was calculated using the following formula: fasting serum insulin (in micro units per milliliter) × fasting serum glucose (in millimoles per liter) divided by the constant 22.5 [28]. The QUICKI was calculated using the following formula: (1/log (fasting insulin) + log (fasting blood glucose) [29].

Statistical analysis

The data obtained were analyzed using SPSS version 26 (IBM Corp., Armonk, NY, USA). The independent t-test was used to compare the means of the two groups of people with T2DM with and without NAFLD, and when normality was not confirmed, the Mann-Whitney U test was used. The chi-square test was used to compare the distribution of categorical variables between the two groups.

The receiver operating characteristics (ROC) curve and the area under the curve (AUC) were used to assess the ability of different indices to identify NAFLD. The optimal cut-off values for the identification of NAFLD were determined from the ROC curve. The association between continuous variables associated with NAFLD was tested with Spearman’s correlation. A p-value <0.05 (two-sided test) was accepted as statistically significant.

## Results

Study participants’ demographic, anthropometric, clinical, and biochemical characteristics

A total of 67 individuals with T2DM were involved in our study, and 28 were diagnosed with NAFLD through ultrasonography. The average age of those without NAFLD was 57 ± 10.6 years, while for those with NAFLD, the mean age was 57.4 ± 11.9 years. The other characteristics of study participants have been reported in our earlier study [13]. Among these participants, there were no significant differences in demographic factors such as age, gender distribution, BMI, and the duration of NAFLD.

However, the anthropometric measurements WC, HC, and WC to height ratio were significantly higher in women with NAFLD than those without (p < 0.001, at least). Hypertension was also more prevalent among those with NAFLD (p < 0.01). Mean HbA1c% or fasting plasma glucose (FPG) was not significantly different between those with and without NAFLD. However, those with NAFLD had significantly higher serum insulin levels (25.9 ± 27.4 vs. 13.1 ± 9.1, p < 0.01). The means (±SD) of newly measured and calculated biomarkers in participants with and without NAFLD and those with abnormal values are presented in Table 1.

**Table 1:** Mean ± SD of fatty liver biomarkers in people with T2DM with and without NAFLD and the number of patients with high biomarker levels. ^: P-value obtained by Mann-Whitney non-parametric test ^~^: P-value obtained by t-test. ^$^: P-value obtained by the chi-square test. Significant p-values are in bold. FFA: free fatty acids; FGF-21: fibroblast growth factor 21; HOMA-IR: homeostatic model assessment of insulin resistance; QUICKI: quantitative insulin sensitivity check index; NAFLD: non-alcoholic fatty liver disease; T2DM: type 2 diabetes mellitus

Fatty liver biomarkers	Participants without NAFLD, N = 39 (men = 16, women = 23)	Participants with NAFLD, N = 28 (men = 6, women = 22)	P-value
FGF-21 (ng/L)			
Mean ± SD (actual range)	113 ± 42 (38–291)	353 ± 168 (143–767)	<0.001^
Number of participants with high FGF-21 (≥166 ng/L) (%)	1 (2.6%)	26 (92.9%)	<0.001^$^
HOMA-IR			
Mean ± SD (actual range)	6.18 ± 5.27 (1.16–22.3)	10.4 ± 10.4 (1.47–45.7)	0.023^
Number of participants with increased insulin resistance (>1.9) (%)	33 (84.6%)	25 (96.2%)	0.142^$^
QUICKI			
Mean ± SD (actual range)	0.309 ± 0.031 (0.253–0.374)	0.291 ± 0.029 (0.234–0.361)	0.018^~^
Number of participants with increased insulin resistance (<0.339) (%)	31 (79.5%)	25 (96.2%)	0.057^$^
FFA			
Mean ± SD (actual range)	0.39 ± 0.18 (0.15–0.85)	0.44 ± 0.11 (0.24–0.74)	0.115^~^

The mean levels of FGF-21 and HOMA-IR were significantly higher, and the mean QUICKI was significantly lower in people with than in those without NAFLD (p < 0.001, p = 0.023, and p = 0.018, respectively (Table 1). High FGF-21 was significantly (p < 0.001) more common among participants with NAFLD (92.9%) compared to those without NAFLD (2.6%) (Table 1).

Predictive ability of FGF-21, HOMA-IR, QUICKI, and FFA for NAFLD

**Figure 1:**
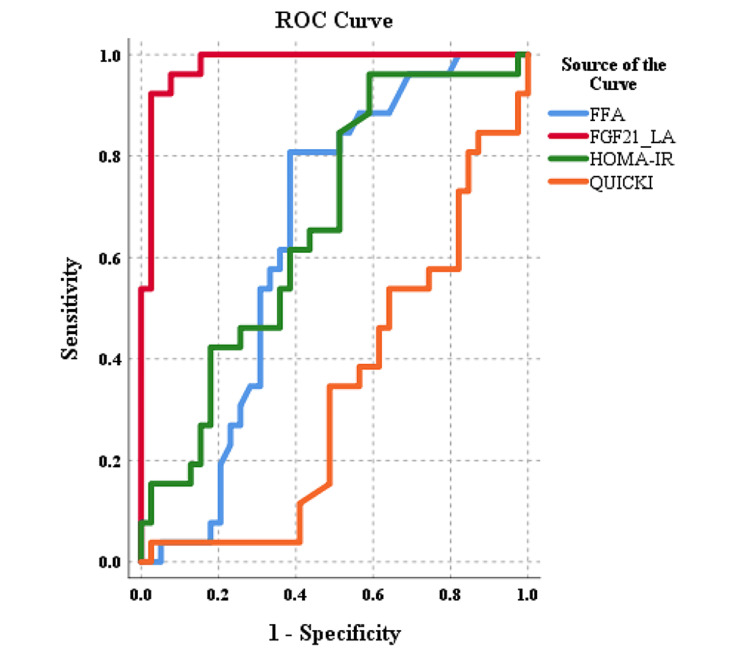
Receiver operating characteristic (ROC) curves. ROC curves for free fatty acids (FFAs), fibroblast growth factor 21 (FGF-21), homeostatic model assessment of insulin resistance (HOMA-IR), quantitative insulin sensitivity check index (QUICKI) in patients with type 2 diabetes.

The ROC curves for the four studied indices are shown in Figure 1. Among all the four indices, FGF-21 had the highest AUC to predict NAFLD (AUC = 0.981, 95% confidence interval = 0.954-1, p < 0.001) (Figure 1 and Table 2). The AUC for HOMA-IR, QUICKI, and FFA were <0.7 (Figure 1 and Table 2).

**Table 2:** Area under the receiver operating characteristic curve (AUC) and its 95% CI for different biomarkers with NAFLD in patients with type 2 diabetes. FFA: free fatty acids; FGF-21: fibroblast growth factor 21; HOMA-IR: homeostatic model assessment of insulin resistance; QUICKI: quantitative insulin sensitivity check index; NAFLD: non-alcoholic fatty liver disease; CI: confidence interval; SE: standard error

Fatty liver biomarkers	AUC	SE	95% CI	P-value
FGF-21	0.981	0.014	0.954, 1	<0.001
HOMA-IR	0.667	0.067	0.535, 0.799	0.023
QUICKI	0.333	0.067	0.201, 0.465	0.023
FFA	0.647	0.069	0.512, 0.782	0.046

The optimal cut-off values for the four investigated biomarkers and calculated sensitivity, specificity, and positive and negative likelihood ratios (PLR and NLR) for predicting NAFLD in people with T2DM using these calculated cut-off values are presented in Table 3. The highest sensitivity, specificity, and PLR combined with the lowest NLR were found when FGF-21 was used to predict NAFLD in people with T2DM (Table 3).

**Table 3:** The optimal cut-off values for fatty liver biomarkers and their sensitivity and specificity for the identification of NAFLD in diabetic patients. FFA: free fatty acids; FGF-21: fibroblast growth factor 21; HOMA-IR: homeostatic model assessment of insulin resistance; QUICKI: quantitative insulin sensitivity check index; PLR: positive likelihood ratio; NLR: negative likelihood ratio; NAFLD: non-alcoholic fatty liver disease

Fatty liver biomarkers	Optimal cut-off point	Sensitivity	Specificity	PLR	NLR
FGF-21	166	0.929	0.974	35.731	0.028
HOMA-IR	3.8	0.846	0.487	1.649	0.606
QUICKI	0.298	0.538	0.359	0.839	1.191
FFA	0.348	0.808	0.615	0.945	0.476

There were no correlations between the level of FGF-21 and the levels of insulin and FPG (data not shown).

## Discussion

Given the high prevalence of T2DM in Saudi Arabia [30,31], the high incidence of NAFLD among T2DM patients [3], and the absence of robust biomarkers for its prediction, the search for biomarkers for this often-silent complication of diabetes is needed to optimize management. Therefore, we aimed to find specific and sensitive markers that may be used to predict NAFLD in Saudi people with T2DM by assessing the most likely candidates, namely, FGF- 21, FFA, and two measures of IR, i.e., HOMA-IR and QUICKI.

In our investigation, the mean levels of FGF-21 and HOMA-IR were significantly higher, while those of QUICKI were significantly lower in people with T2DM with NAFLD compared with those without NAFLD. In addition, FGF-21 had the highest AUC to predict NAFLD compared with the other three indices, with a cut-off value of 166 ng/L showing high sensitivity, specificity, and PLR combined with a low NLR.

We reported earlier [13] that the mean levels of HbA1c% or FPG were not significantly different between those with and without NAFLD, but those with NAFLD had significantly higher serum insulin levels. Therefore, the means of the two calculated measures of IR (HOMA-IR and QUICKI) were significantly different in the group of patients with NAFLD compared with the means of the group without the condition, indicating an association between increased IR and NAFLD in people with T2DM.

IR has long been proposed as the first hit in the two-hit hypothesis [32], which explains the pathogenesis of NASH, with excessive fatty acids in circulation leading to simple hepatic steatosis and IR, thus promoting the progression from simple fatty liver to NASH [33]. Therefore, we studied measures of IR as biomarkers for predicting NAFLD in Saudi people with T2DM. However, following rigorous statistical analysis, the AUC for HOMA-IR and QUICKI were both <0.7, which is reflected in low specificity and unacceptable PLR and NLR for both calculated measures. Therefore, it can be suggested that neither of these measures can be used to predict NAFLD in our T2DM patients.

The use of HOMA-IR and other IR markers in NAFLD has been investigated by different research groups, particularly among non-diabetic populations. A recent study conducted on 2,148 non-diabetic Chinese adults between 2021 and 2023 found that HOMA-IR along with TyG (triglyceride-glucose index), TyG-BMI (TyG multiplied by body mass index), TG/HDL-c (triglycerides to high-density lipoprotein cholesterol ratio), and METS-IR (metabolic score for insulin resistance) were effective in predicting the risk of NAFLD. However, the study highlighted that the predictive abilities of these IR markers varied between obese and non-obese populations, with HOMA-IR showing significant predictive value in obese populations for NAFLD [34].

Another published study by the same group included 2,234 participants recruited between 2021 and 22 and investigated the correlation between HOMA-IR and NAFLD in a non-diabetic Chinese population. Similar to our findings, HOMA-IR was significantly higher in the NAFLD group. The efficacy of HOMA-IR for diagnosing NAFLD was substantiated using ROC curves, demonstrating its utility as a predictive tool for NAFLD in lean, non-diabetic Chinese individuals in contrast to our findings [35].

A prospective, cohort, population-based study was conducted in the northern region of Iran among 2,461 participants without NAFLD (with and without diabetes) between 2009 and 2010, who were recruited using the stratified randomization method based on the sex and age of individuals. Ultrasonographic examination was performed at the baseline and after a seven-year follow-up between 2016 and 2017. Multiple binary regression analysis was applied to evaluate the association between the development of NAFLD and potential risk factors. Based on the numerous binary logistic regression analyses, HOMA-IR had a significant relationship with the incidence of NAFLD in women (odds ratio = 1.164, 95% confidence interval = 1.041-1.301, p = 0.007] but not in men, concluding that HOMA-IR can be considered an independent risk factor for NAFLD in women only [36].

In an earlier published study, the same researchers using the baseline data of the cohort mentioned above and collected from 5,511 participants with and without NAFLD (diabetic and non-diabetic) aged ≥18 years determined the optimal cut-off points for HOMA-IR and QUICKI in the diagnosis of MetS and NAFLD. The optimal cut-off point of HOMA-IR for the diagnosis of NAFLD was 1.79 (sensitivity = 66.2%, specificity = 62.2%) in men and 1.95 (sensitivity = 65.1%, specificity = 54.7%) in women. In addition, the optimal cut-off point of QUICKI for the diagnosis of NAFLD was 0.347 (sensitivity = 62.9%, specificity = 65.0%) in men and 0.333 (sensitivity = 53.2%, specificity = 67.7%) in women, concluding that the optimal cut-off points of HOMA-IR and QUICKI were different for men and women [37]. However, unlike our study, they did not report PLR and NLR, which are necessary to decide whether the investigated biomarker is robust enough to be used to predict a disease.

Gender differences were not investigated in our study due to the small number of included participants. The noted differences between our findings and the above-mentioned studies could be due to differences in ethnicities, the demographic and clinical characteristics of the studied population, and the differences in lifestyle and dietary practices.

Another investigated biomarker in our study was the level of circulating FFAs. As mentioned earlier, an increased level of circulating FFAs has been reported to be associated with increased lipolysis and FFA flux to the liver, leading to increased synthesis of triglycerides [26]. We found no difference in the mean levels of FFAs between participants with and without NAFLD, and the calculated AUC was <0.7. However, using the optimal cut-off value of 0.348, the circulating level of FFAs showed a reasonable sensitivity of 0.808 and a moderate specificity of 0.615. Despite this, the calculated PLR and NLR ruled out the possibility of using FFAs as a predictive marker for NAFLD in Saudi people with T2DM.

A cross-sectional Chinese study, which included 840 participants with NAFLD (114 diabetics and 726 non-diabetics) and 331 healthy controls, investigated the association between fasting serum FFAs and NAFLD. Serum FFA levels were significantly higher in people with NAFLD compared to controls (p < 0.001), and stepwise regression indicated that the level of serum FFAs was an independent factor predicting advanced fibrosis (Fibrosis-4 (FIB-4) ≥1.3) only so that it could be used as an indicator for predicting advanced fibrosis, but not milder fibrosis (FIB-4 <1.3) in NAFLD patients [38]. We did not measure the degree of fibrosis in our study, and all included patients were diabetic, which could explain the difference between our findings and those of the Chinese study.

Another study evaluated FFA profiles among healthy Chinese individuals and NAFLD patients (lean, overweight, and obese) to identify the most likely FFAs that can be used for the early diagnosis of NAFLD. The serum FFA profiles of NAFLD patients were significantly higher compared to healthy controls. Furthermore, there was no significant difference in total FFA profiles between lean and overweight NAFLD patients. In contrast, the total FFA profiles of obese NAFLD patients were significantly higher compared with the profiles of healthy controls and lean and overweight NAFLD patients. However, following rigorous statistical analysis and adjusting for confounding factors, it was concluded that only myristic acid (14:0) and palmitoleic acid (16:1), and not total FFA, can be considered promising for the early diagnosis of NAFLD, especially among normal-weight individuals [39]. We estimated the total FFA in our study, ruling out its usefulness in predicting NAFLD, and substantiated their findings.

As mentioned earlier, the development of NAFLD has been reported to be associated with various causes [21-25]. In addition, dietary energy intake and diet composition have also been shown to play an important role [40]. High-fat diets have been reported to cause fatty liver [41]. Therefore, it can be suggested that an increase in de novo lipogenesis due to dietary intake or other causes can be the leading cause of accumulation of fat in the liver of our studied patients, hence, the lack of difference in the mean levels of FFAs between the two groups of patients with and without NAFLD, especially as both studied groups were T2DM patients with various degrees of IR. This suggestion requires further research which should include dietary intake studies and perhaps the inclusion of non-diabetic individuals with NAFLD to clarify the situation and explore the possibility of utilizing FFAs to diagnose NAFLD in non-diabetic people.

Our fourth investigated biomarker was the serum level of FGF-21. This showed very promising results for diagnosing NAFLD with very high specificity and sensitivity, giving a high PLR and low NLR. Therefore, serum FGF-21 ≥166 ng/L could be suggested as a good predictor for the diagnoses of NAFLD in Saudi T2DM patients.

In partial agreement with our study, an earlier study among 179 Chinese NAFLD patients with and without diabetes (68 NASH cases and 111 non-NASH cases) reported that the serum levels of several biomarkers, including FGF-21, were significantly higher in NAFLD patients compared with healthy controls. In addition, these levels positively correlated with NAFLD activity scores (NAS) and pathological characteristics of NAFLD, concluding that these biomarkers could be non-invasive diagnostic markers for NASH, especially if measured in a stepwise combination [42]. When measuring FGF-21 alone to diagnose NASH, their reported sensitivity was 79.30% with a specificity of 77.40%, which is lower than our findings. The difference between our results and those of the aforementioned study could be due to differences in the clinical characteristics of included participants (T2DM patients versus mixed population), ethnicity, genetics, and dietary practices, especially as dietary intake and composition have been shown to play a considerable role in the development of NAFLD as mentioned earlier [41], and there have been reports of ethnicity-associated differences in the pathogenesis and development of NAFLD [43]. Furthermore, a study among morbidly obese females undergoing bariatric surgery did not show any association between pathological features of NASH and plasma FGF-21, which suggests that body fat and gender, as well as other comorbidities, may modify this association [44].

As in most studies, our study has limitations and strengths. The main limitation of our study was the small sample size, which did not allow the investigation of the effect of gender differences, especially as the liver shows a very high degree of sexual dimorphism [45,46]. A second limitation is that this study did not estimate the degree of steatosis, which could affect the level of measured markers. Another limitation was that dietary intake was not studied, even though it might affect the development of the condition and the level of FGF-21. However, as the first study on Saudi T2DM patients, our study shows a strong relationship between NAFLD and the level of FGF-21, suggesting its use as a possible biomarker for predicting NAFLD, thus helping to plan future research. Further studies, including more novel biomarkers on a larger population from different parts of Saudi Arabia, are needed to validate our findings. In addition, studies on non-diabetic individuals are also required and are planned, once funding is available, to explore the possibility of using novel biomarkers to predict NAFLD in these individuals.

## Conclusions

Our research indicates that the serum level of FGF-21 is a highly specific and sensitive biomarker for predicting NAFLD in Saudi patients with T2DM. This biomarker could be utilized for preliminary screening before patients are referred to more advanced healthcare facilities for confirmatory tests such as abdominal ultrasonography. Although HOMA-IR and QUICKI are associated with NAFLD, their predictive power is limited. Additionally, the study found no significant difference in FFA levels between the groups, suggesting that FFAs are not effective in predicting NAFLD.
